# Regulation of tumor antigens-Dependent immunotherapy via the hybrid M1 macrophage/tumor lysates Hydrogel

**DOI:** 10.1016/j.heliyon.2024.e37521

**Published:** 2024-09-06

**Authors:** Zeyang Li, Jiani Zhan, Yinuo Zheng, Yingli Luo, Xiaoming Yu, Haha Chen

**Affiliations:** a Department of Ultrasonic Imaging, The First Affiliated Hospital of Wenzhou Medical University, Wenzhou, 325000, Zhejiang, China; b The First School of Medicine, School of Information and Engineering, Wenzhou Medical University, Wenzhou, 325000, Zhejiang, China; c Department of Thyroid and Breast Surgery, Oncological Surgery, Ruian People's Hospital, The Third Affiliated Hospital of Wenzhou Medical University, Wenzhou, 325200, Zhejiang, China; d Wuxi School of Medicine, Jiangnan University, Wuxi, 214122, Jiangsu, China; e Cancer Center, Department of Pulmonary and Critical Care Medicine, Zhejiang Provincial People's Hospital, Affiliated People's Hospital, Hangzhou Medical College, Hangzhou, 310014, Zhejiang, China

**Keywords:** Tumor immunotherapy, M1/M2 macrophages, Hybrid hydrogel, Antigens presentation

## Abstract

Tumor treatment poses a significant obstacle in contemporary healthcare. Using components derived from a patient's own cellular and tissue materials to prepare hydrogels and other therapeutic systems has become a novel therapeutic approach, drawing considerable interest for their applicability in basic research on cancer immunotherapy. These hydrogels can engage with cellular components directly and offer a supportive scaffold, aiding in the normalization of tumor tissues. Additionally, their superior capability for encapsulating targeted anti-tumor medications amplifies treatment effectiveness. Given their origin from a patient's own cells, these hydrogels circumvent the risks of immune rejection by the body and severe side effects typically associated with foreign substance. In this study, we developed a composite hydrogel constructed by the cellular lysates of autologous tumor cells and M1 macrophages. This combination promoted the M2 macrophages polarization to the M1 phenotype. Subsequently, the polarized M1 macrophages infiltrated into the hydrogel and can directly capture tumor antigens. As antigen-presenting cells, M1 macrophages can stimulate the production of antigen-specific T cells to kill tumor cells. This work proposes a dual-benefit research strategy that not only polarizes M2 macrophages but also enhances immune activation, boosting T cell-mediated tumor-killing effects. This approach offers a new therapeutic option for clinical cancer immunotherapy.

## Graphical abstract

The illustration presents a hydrogel formulation created through the amalgamation of lysates from both tumor cells and M1 macrophages. This innovative hydrogel setup successfully facilitates the conversion of M2 macrophages to M1 macrophages and achieves the presentation of tumor antigens, thereby triggering the activation of T cells specific to these antigens.

**Figure:**
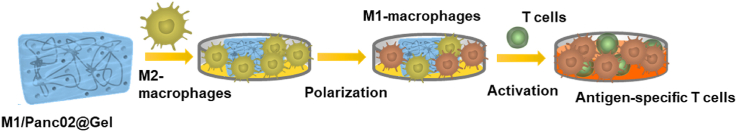

## 1 Introduction

Water-based hydrogels are emerging as versatile tools in oncological treatments, showing promise across various aspects of cancer therapy [1]. These hydrogels, consisting of a hydrophilic polymer network that retains a substantial amount of water, possess distinct characteristics ideal for diverse therapeutic modalities [2]. Specifically, in cancer care, their utility in the precise delivery of therapeutic agents to malignancies has been a subject of considerable research interest [3]. Encapsulating chemotherapeutic or immunomodulatory substances within these hydrogels facilitates a controlled and sustained release, targeting the medication directly to tumor sites [4]. Such localized delivery reduces systemic side effects and boosts the effectiveness of the treatments [5].

Beyond drug delivery, hydrogels serve as excellent scaffolds for regenerating tissue damaged by tumors or their removal [6]. They simulate the natural extracellular matrix, providing a conducive environment for cellular growth and organization [7]. This capability is instrumental in reconstructing tissues post-surgery and creating in vitro tumor models for drug testing and tailored therapies [8]. Moreover, hydrogels designed to react to specific environmental stimuli, like pH, temperature, or enzymatic activities [9], have paved the way for developing 'smart' hydrogels. These materials release their therapeutic cargo in response to specific tumor-associated triggers [10], enhancing treatment selectivity and minimizing non-targeted effects [10]. Recently, hydrogels have been widely used in the stimulation of anti-tumors immunotherapy [11], encapsuling immune response-activating substances or tumor associated antigens to stimulate immune infiltration into the tumor microenvironment [12]. This strategy shows promise in boosting the effectiveness of immunotherapy and further combating immunosuppression tumor environment [13]. Although the advances, hydrogels applied in the clinic tumor therapy have a lot of hurdles [14], such as biocompatibility, degradation rates, and large-scale production [15].

Hydrogels derived from autologous tissues or cells present a novel approach in cancer therapy, offering personalized, biocompatible platforms for a range of therapeutic strategies [16,17]. The primary advantage of autologous hydrogels lies in their exceptional biocompatibility [18]. Originating from the patient's own biological material, these hydrogels significantly reduce the occurrence rate of adverse reactions [19], ensuring safer, long-term applications [20]. In tumor therapy, hydrogels derived from autologous cells have shown promise about the localized drug delivery [21]. By integrating anti-tumor agents or immunotherapeutic substances into the hydrogel system, a localized, controlled release system is completed [22]. The method enhances treatment efficacy by directing therapeutics precisely to the tumor site while minimizing systemic exposure [23]. Furthermore, these autologous cells derived hydrogels can be tailored easily to simulate extracellular matrix [24], playing a vital role in cell signaling and migration [25]. Creating a biomimetic scaffold with autologous hydrogels supports cellular architecture and growth, aiding tissue regeneration following tumor excision [26], an essential aspect of reconstructive surgery and tissue engineering [27].

In immunotherapy, autologous hydrogels loaded with immune-boosting molecules or tumor antigens have shown potential in activating the body's immune defense against tumors [[28], [29]]. This approach enhances the infiltration of immune cells and strengthens anti-tumor immunity, offering a promising avenue to augment immunotherapy outcomes and disrupt the immunosuppressive tumor milieu [30]. Nonetheless, translating autologous hydrogels into clinical practice faces challenges, including scalability, cost, and complex manufacturing processes [31]. Yet, with continued research and technological progress, these barriers are increasingly being addressed [32].

This investigation introduces a novel strategy employing hybrid hydrogels from autologous sources to reprogram the tumor immune microenvironment. Combining lysates from M1 macrophages and tumor cells, we formulate a hybrid hydrogel. This gel is designed to transform M2 macrophages prevalent in tumor tissues into the M1 phenotype, leveraging the antigen-presenting properties of M1 macrophages to stimulate T-cell-mediated tumor clearance. Our findings indicate that this hybrid hydrogel effectively repolarizes M2 macrophages to M1, enhances antigen presentation by M1 macrophages, and activates T cells, offering a promising new direction in tumor immunotherapy.

## 2 Results and discussion

**Fig. 1:**
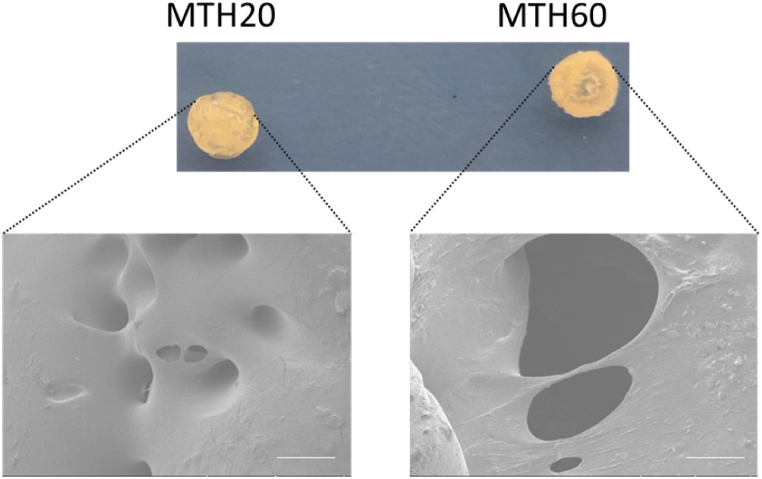
Synthesis and structural analysis of hybrid bioactive cell lysate hydrogel. MTH20 and MTH60 (diameter of 5.5 mm and height of 3.5 mm) denote hydrogel constructs formulated using M1 macrophage to Panc02 cell lysate ratios of 1:4 and 3:2, correspondingly (scale bar: 100 μm).

To fabricate the composite hydrogel, cellular lysates of M1 phenotype macrophages and the pancreatic cancer cell line (Panc02 cells) were isolated and further combined at varying proportions, leading to the formation of three-dimensional hydrogel constructs. Two specific mix ratios, based on the concentration of proteins, were selected: a 1:4 ratio of M1 macrophage to Panc02 cell lysates for one batch, and a 3:2 ratio for another, subsequently referred to as MTH20 and MTH60, respectively. The hydrogelation process was initiated through the addition of NHS and EDC as catalysts at a temperature of −20 °C, facilitating the development of robust three-dimensional hydrogel matrices. The physical attributes and dimensions of the resulting hydrogels were modifiable under different experimental setups. Additionally, examination via scanning electron microscopy disclosed a porous architecture within both hydrogel variants, with a notable distinction in the pore dimensions observed between them. Specifically, MTH60 exhibited a significantly enlarged pore size compared to MTH20 (Fig. 1). Such variations in the internal structure suggest disparities in biological function and inherent properties between the two hydrogel formulations.

**Fig. 2:**
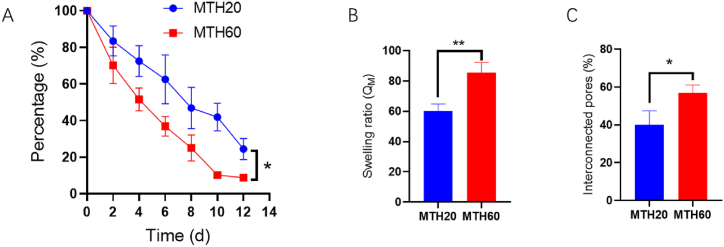
Degradation rates (A), absorbency levels (B), and pore structure metrics (C) for the two distinct hydrogel formulations (*P < 0.05, **P < 0.01).

To elucidate the disparities in the physical characteristics of hydrogels derived from two distinct cell lysate mixtures, an initial assessment of their degradation behavior was conducted. Cylindrical specimens of the hydrogels, each measuring 2.5 cm in diameter and 1 cm in height, were submerged in 2 mL of deionized water. At designated intervals, these specimens were retrieved, subjected to lyophilization, and their mass was recorded to gauge the degradation kinetics. As depicted in Fig. 2A, MTH60 showed a marginally accelerated degradation pace in comparison to MTH20. Nonetheless, both variants underwent degradation over a span of 12 days, indicating their capability to sustain functionality over an extended period post in vivo application. Further analyses focused on evaluating the swelling behavior and porosity of the two hydrogel versions. While the variances observed were minimal, MTH60 demonstrated enhanced swelling capacity and a greater porosity index (as shown in Fig. 2B and C). Coupled with its quicker degradation rate and enlarged pore dimensions, MTH60's attributes suggest it may offer distinct biological advantages.

**Fig. 3:**
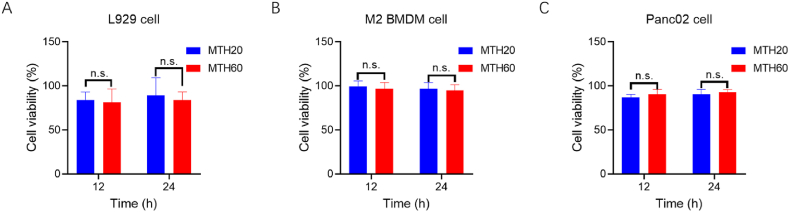
The determined cell viability of L929 cells (A), M2 macrophages (B), and Panc02 cells (C) treated with the different hydrogel's formulations (n.s. = no signification).

To substantiate the hydrogels' biological effectiveness and their capability to modulate cellular behaviors, cytotoxicity evaluations were performed on relevant cell models. L929 cells as non-tumor cells representatives, pancreatic cancer cells of Panc02 cells, and the collected M2 macrophages in the tumor environments were chosen for these assays to mimic the primary cellular interactions expected in vivo with the hydrogels. These cell types were cultured in 24-well plates with the hydrogels introduced into adjacent Transwell chambers. The setup was incubated under a CO_2_ atmosphere for durations of 12 or 24 h, after which the cells viability were determined with the usage of CCK-8 kits. Observations recorded at both intervals indicated an absence of notable cytotoxicity from the hydrogels towards the cells, with all cell types exhibiting sustained viability (Fig. 3A–C). This evidence underscores the hydrogels' compatibility and suggests their safety for potential in vivo utilization.

**Fig. 4:**
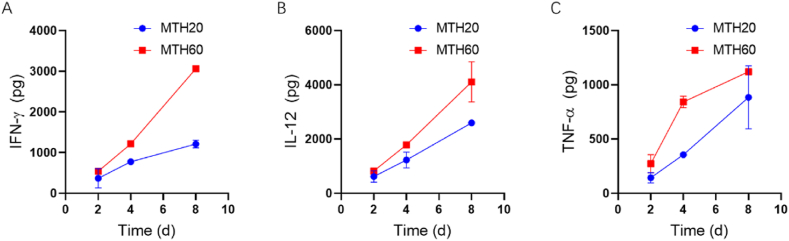
The releasement kinetics of IFN-γ (A), IL-12 (B), and TNF-α (C) in MTH20 or MTH60.

Following the verification that the hydrogels did not induce marked cytotoxic effects on cells, we next explored the profiles of regulatory molecules present within the hydrogels, focusing on the cytokines’ releasement dynamics. Hydrogels of different formulations were directly immersed into the 500 μL distilled water, and then the samples were periodically extracted to quantify the content of certain critical cytokines, such as TNF-α, IL-12, and IFN-γ, associated with M1 macrophages through enzyme-linked immunosorbent assay (ELISA) tests. These cytokines are pivotal for the transition of M2 macrophages to M1 macrophages, a process integral to the hydrogels' intended bioactive role. The measurements, depicted in Fig. 4A–C, reveal that initially, over the first 48 h, the cytokine release rates from both hydrogel versions were comparably modest. Yet, with the passage of time, a parallel trend in the release patterns for all three cytokines was noted. Remarkably, MTH60 demonstrated a more pronounced release of these regulatory factors compared to MTH20, suggesting that MTH60 harbors enhanced bioactive capabilities and shows more promise in driving the polarization of M2 macrophages.

**Fig. 5:**
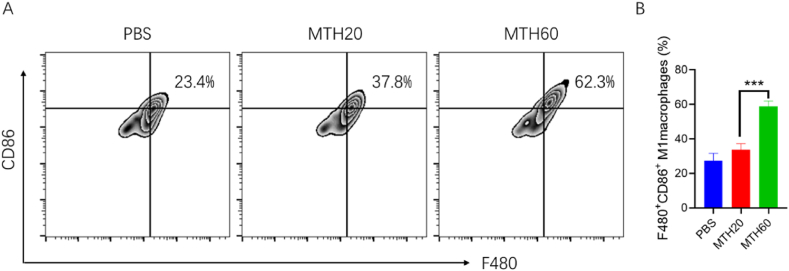
Flow cytometry analysis of M1 phenotype macrophages (A) and statistical analysis (B) after the treatment of M2 phenotype macrophages with the MTH20 or MTH60 (***P < 0.005).

To evaluate the capacity of the two hydrogels to induce polarization in M2 phenotype macrophages collected from the tumor environments, the cells were incubated with the prapared hydrogels. Subsequently, macrophages were collected, and the shift towards M1 macrophages was quantified using flow cytometry. A notable augmentation in M1 macrophage percentages was detected in samples exposed to both hydrogels. Notably, cells treated with MTH60 exhibited an approximately threefold elevation in M1 macrophage levels in comparison to the untreated control group (Fig. 5A and B). These findings underscore the potent polarization-inducing effect of MTH60 hydrogel on M2 to M1 macrophage transformation. Such results hint at the potential of employing MTH60 in transforming immunologically "cold" tumors into "hot" ones, potentially boosting the efficiency of immunotherapeutic approaches against tumors.

**Fig. 6:**
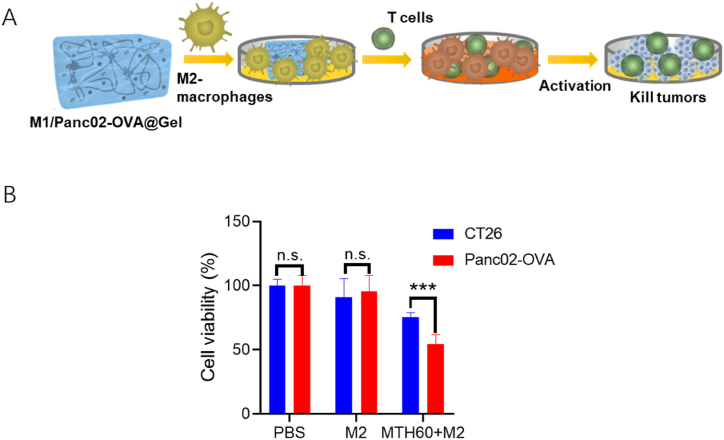
The strategy of M2 phenotype macrophages induced via the prepared hydrogel and then stimulation of T cells to inhibit the tumor growth (A). The inhibition of different tumor cells via the hydrogel of MTH60 (B) (***P < 0.005, ns = no signification).

The obtained data from our experiments affirm that the engineered hydrogel effectively drives the conversion of M2 macrophages to M1 macrophages, with the MTH60 formulation showcasing superior efficacy. Given the pivotal function of M1 macrophages in antigen processing and presentation, the inclusion of tumor cell lysate within the hydrogel acts as a potent antigen reservoir. This arrangement promotes antigen assimilation and presentation by M1 macrophages, ultimately leading to the activation of specific T lymphocytes targeted towards tumor eradication (Fig. 6A). To delve deeper into the capacity of M1 phenotype macrophages to present tumor antigens and to ascertain if the activated T cells possess tumor-specific cytotoxicity, a sequential co-culture was established. Initially, M2 macrophages were exposed to the MTH60 hydrogel, followed by their interaction with naive T cells freshly isolated from the organism. Subsequently, these primed T cells were exposed to the CT26 or the Panc02 tumor cells to evaluate the cytotoxic potential against the different cancer cells. As depicted in Fig. 6B, T cells without MTH60 exposure demonstrated negligible cytotoxic activity towards both cancer cell types. Conversely, T cells primed with MTH60 showed pronounced cytotoxicity against Panc02 cells but minimal effect on CT26 cells. These observations underscore MTH60's ability to not only stimulate M2 macrophage polarization but also to enable effective tumor antigen presentation within the hydrogel. This process culminates in the generation of T cells specifically primed to target and eliminate tumor cells selectively.

## 3 Conclusion

Hydrogels of 3D structures were made of various hydrophilic polymers, which have garnered significant interest for the cancer treatment applications. Specifically, hydrogels synthesized from autologous cells are becoming increasingly popular due to their exceptional biocompatibility and their capacity to replicate the natural tissue microenvironment. Hydrogels fabricated from the cells of patients, which have demonstrated a tailored and individualized therapeutic modality. Recent advancements in the field have seen the use of autologous cell-based hydrogels as vehicles for delivering a variety of therapeutic agents, such as chemotherapy drugs, genetic therapy agents, and immune checkpoint blockers. Furthermore, efforts have been focused on enhancing the immunomodulatory properties of these hydrogels to recruit and stimulate immune cells, with the goal of achieving targeted tumor cell destruction.

In our research, we developed a novel hybrid hydrogel by combining the tumor cellular lysates and the M1 macrophages lysates. Our findings indicate that this hydrogel prompts a shift in M2 macrophages towards an M1 phenotype, effectively altering the tumor's immunosuppressive environment, predominantly composed of pro-tumorigenic M2 macrophages, to a more immunologically active "hot" tumor scenario. Moreover, M1 macrophages, acting as proficient antigen-presenting cells, are capable of processing and presenting tumor antigens. Subsequent experiments revealed that these M1-polarized macrophages could successfully present tumor antigens, leading to the activation of T cells specific to these antigens, which then specifically target and eliminate tumor cells. The outcomes of our investigation suggest that the hydrogel fabricated by the two different cell lysates could act as a promising platform for tumor immunotherapy, potentially revolutionizing personalized cancer treatment strategies.

## 4 Materials and methods

### 4.1 Synthesis of Hydrogel developed by the different cell lysate

To procure the different cell lysates, cells from M1 macrophages and Panc02 cancer line were disrupted using a standard cell lysis solution available in the market. The process involved a 30-min incubation with the lysis buffer on ice, succeeded by centrifugation to discard cell remnants. We describe a method to create the novel hydrogel by blending the cell lysate in precise ratios, adding a specific volume of N-hydroxysuccinimide (NHS) and 1-ethyl-3-(3-dimethylaminopropyl) carbodiimide hydrochloride (EDC) to the mix. The solutions underwent vigorous shaking before being cooled at −20 °C, resulting in the formation of a hydrophilic gel.

### 4.2 Analysis of the hybrid Hydrogel Features

Degradation profile: The mass of samples was monitored over time during degradation. The percentage of mass loss is evaluated by contrasting the initial weight with the weight recorded at subsequent intervals.

Swelling behavior: Hydrogel specimens are immersed into the specified buffer or solvent, with the slow increase in the weight. The swelling index was calculated by the ratio of the increased weight to the untreated weight.

### 4.3 Evaluating Hydrogel cytotoxicity

The cytotoxic impact of the prepared hydrogel was crucial for the application in clinical tumor therapy. The CCK-8 assay, relying on the content of tetrazolium salt to gauge cell viability, is employed for assessing the viability of different cells exposed to hydrogel. Post 12 or 24 h of hydrogel exposure, CCK-8 solution was added into the cells, and the absorbance at 405 nm is measured by the plate reader machine. The number of absorbance readings were utilized for the calculation of cell viability, expressed as the percentage compared to untreated controls.

### 4.4 Identifying cytokine release from hydrogels

Hydrogel samples are incubated in conditions that simulate the in vivo environment, using cell culture media. The supernatant is collected after a set period to gather cytokines released from the hydrogel. Levels of IFN-γ, IL-12, and TNF-α were further measured through ELISA, with statistical analysis comparing cytokine levels across different hydrogel formulations to ascertain their immune-modifying capabilities.

### 4.5 Assessing M2 to M1 macrophage polarization induced by hydrogels

The tumor tissue were obtained and cut into small pieces (1–2 mm³), and then they were placed into a digestion solution containing enzymes (collagenase, trypsin, and DNase) and incubated at 37 °C for 1 h, gently agitating to facilitate digestion. The digestion solution were filtered through a cell strainer to remove undigested tissue fragments. The cells were washed multiple times with sterile PBS (phosphate-buffered saline) or culture medium to remove enzymes and debris. The cell suspension were centrifuged at low speed (350 g, 10 min) to collect the cell pellet. Subsequently, the cells were resuspended in fresh PBS or an appropriate culture medium. The cells were incubated with fluorescently labeled antibodies (CD206-FITC) for 45 min at 4 °C, protected from light. The labeled cells were run through a flow cytometer to sort M2 macrophages based on surface markers. Then, M2 macrophages sourced from tumor tissues are treated with hydrogel extracts, promoting their polarization towards an M1 phenotype over 48 h. Post-treatment, macrophages are stained with antibodies targeting M1 markers (F480 and CD86) and analyzed via flow cytometry to quantify the polarization effect.

### 4.6 T cell activation and targeted tumor cell Elimination by polarized M1 macrophages

Hydrogels based on Panc02 cells and M1 macrophages are synthesized. M2 phenotype macrophages, collected from the mice of Panc02-OVA tumors, were then treated with the hydrogel, inducing a shift from the M2 to the M1 phenotype macrophages. These M1 phenotype macrophages, loaded with tumor antigens from the hydrogel, were incubated with special T cells isolated from OT-1 mice. The ability of these activated T cells to specifically target and kill tumor cells of Panc02-OVA is subsequently evaluated, with statistical methods used to assess the hydrogel's efficacy in facilitating T cell-mediated tumor destruction.

### 4.7 Statistical evaluation

Experiments were replicated across three separate hydrogel batches. Variability in the data is represented by error bars, reflecting the triple determination across different samples.

## Ethical statement

Not applicable.

## Ethics approval and consent to participate

Not applicable.

## Consent for publication

All authors agreed with the content and give their consent to submit the manuscript for publication.

## Funding

Not applicable.

## Data availability statement

The datasets generated during and/or analyzed during the current study are available from the corresponding author on request.

## CRediT authorship contribution statement

**Zeyang Li:** Software, Project administration, Methodology, Data curation. **Jiani Zhan:** Visualization, Validation, Resources, Investigation. **Yinuo Zheng:** Software, Resources, Methodology, Formal analysis. **Yingli Luo:** Writing – review & editing, Writing – original draft, Validation, Software, Methodology, Investigation. **Xiaoming Yu:** Writing – original draft, Supervision, Methodology, Investigation. **Haha Chen:** Writing – review & editing, Writing – original draft, Supervision, Software, Methodology, Investigation.

## Declaration of competing interest

The authors declare that they have no known competing financial interests or personal relationships that could have appeared to influence the work reported in this paper.
